# Organic Matter Responses to Radiation under Lunar Conditions

**DOI:** 10.1089/ast.2015.1442

**Published:** 2016-11-01

**Authors:** Richard Matthewman, Ian A. Crawford, Adrian P. Jones, Katherine H. Joy, Mark A. Sephton

**Affiliations:** ^1^ Impacts and Astromaterials Research Centre, Department of Earth Science and Engineering, Imperial College London, London, UK.; ^2^ Department of Earth and Planetary Sciences, Birkbeck College, University of London, London, UK.; ^3^ Department of Earth Sciences, University College London, London, UK.; ^4^ School of Earth, Atmospheric and Environmental Sciences, University of Manchester, Manchester, UK.

## Abstract

Large bodies, such as the Moon, that have remained relatively unaltered for long periods of time have the potential to preserve a record of organic chemical processes from early in the history of the Solar System. A record of volatiles and impactors may be preserved in buried lunar regolith layers that have been capped by protective lava flows. Of particular interest is the possible preservation of prebiotic organic materials delivered by ejected fragments of other bodies, including those originating from the surface of early Earth. Lava flow layers would shield the underlying regolith and any carbon-bearing materials within them from most of the effects of space weathering, but the encapsulated organic materials would still be subject to irradiation before they were buried by regolith formation and capped with lava.

We have performed a study to simulate the effects of solar radiation on a variety of organic materials mixed with lunar and meteorite analog substrates. A fluence of ∼3 × 10^13^ protons cm^−2^ at 4–13 MeV, intended to be representative of solar energetic particles, has little detectable effect on low-molecular-weight (≤C_30_) hydrocarbon structures that can be used to indicate biological activity (biomarkers) or the high-molecular-weight hydrocarbon polymer poly(styrene-co-divinylbenzene), and has little apparent effect on a selection of amino acids (≤C_9_). Inevitably, more lengthy durations of exposure to solar energetic particles may have more deleterious effects, and rapid burial and encapsulation will always be more favorable to organic preservation. Our data indicate that biomarker compounds that may be used to infer biological activity on their parent planet can be relatively resistant to the effects of radiation and may have a high preservation potential in paleoregolith layers on the Moon. Key Words: Radiation—Moon—Regolith—Amino acids—Biomarkers. Astrobiology 16, 900–912.

## 1. Introduction

Active organic chemistry is ubiquitous throughout the Solar System. From the hydrocarbon lakes of Titan to volatile-rich comets and asteroids, organic matter is continually being processed and redistributed. The evolution of organic chemical systems in the early Solar System ultimately led to the origin of life on Earth. Some of the prebiotic organic matter may have been generated on Earth itself by a variety of mechanisms. These include formation from gases in the atmosphere through electrical discharges or ultraviolet light, or processing by impact shocks (Chyba and Sagan, 1992), and possible abiotic synthesis at hydrothermal vents (McDermott *et al.,* 2015). In addition, a substantial proportion of Earth's prebiotic organic material could have been delivered by small carbonaceous bodies such as asteroids and comets. These bodies can contain significant quantities of organic material (Ehrenfreund and Charnley, 2000; Sephton, 2002; Capaccioni *et al.,* 2015).

There was large-scale delivery of asteroidal and cometary material to Earth during a period approximately 4 billion years ago known as the Late Heavy Bombardment (LHB) (Gomes *et al.,* 2005; Bottke *et al.,* 2012; Marchi *et al.,* 2014). There was substantial contribution from small particles as well as large impactors. Court and Sephton (2014) estimated the flux of micrometeorites to Earth during the peak 50 Myr of the LHB as 8.4 (±4.7) × 10^13^ g yr^−1^, substantially higher than the present-day estimated infall rate of 4 (±2) × 10^10^ g yr^−1^ (Love and Brownlee, 1993; Court and Sephton, 2014). Estimates for the total amount of material delivered to the Moon during the LHB are on the order of 6 × 10^21^ g (Levison *et al.,* 2001; Kring and Cohen, 2002; Gomes *et al.,* 2005). The larger gravitational attraction of Earth would have resulted in a substantially higher number of asteroid impacts on Earth than on the Moon (Bottke *et al.,* 2012). However, the impact record on Earth from this time no longer exists. Plate tectonics and erosion have destroyed rocks older than ∼3.8 Ga that might have preserved some of this information, denying us direct knowledge of the terrestrial prebiotic environment. Examination of asteroidal samples has given us a great deal of information about some of the organic starting materials, but this cannot provide a chronology of the types of impactor that were delivering material to the surface of Earth or tell us how this material was subsequently processed. There remains a potential opportunity to delve into the impact record of ancient Earth by looking at the Moon, where asteroidal (Joy *et al.,* 2012) and cometary (Zhang and Paige, 2009; Anand, 2010; Ong *et al.,* 2010) material could be preserved. The potential for such a mode of preservation on the Moon is illustrated by the discovery of the hydrated carbonaceous chondrite meteorite “Bench Crater,” which was identified within samples collected during the Apollo 12 mission (McSween, 1976; Zolensky, 1997).

In addition to asteroidal and cometary material, rocks ejected from early Earth by impacts may also be preserved on the Moon (Armstrong *et al.,* 2002; Crawford *et al.,* 2008). Modeling work has shown that large impacts on Earth's surface could have ejected fragments that then landed on the Moon (Armstrong *et al.,* 2002; Armstrong, 2010). It has been shown that shock effects for meteorites during ejection from Earth and impact on the Moon are in the range within which organic material could have survived (Crawford *et al.,* 2008; Parnell *et al.,* 2010; Burchell *et al.,* 2014). The impact survival of organic structures is of particular significance as it raises the possibility that biotic and prebiotic organic molecules contemporaneous with life's development on Earth could be preserved on the Moon, providing access to a record that has been completely lost on Earth.

Organic matter that has been delivered to the lunar surface may be destroyed by physical impacts and radiation. These processes constitute “space weathering” and can cause substantial alteration to materials directly exposed at the surface. Micrometeorites impact the lunar surface at high velocities, causing pitting and vaporization of the impactor and target materials (Keller and McKay, 1997). Radiation from solar ultraviolet light, the solar wind, energetic solar events (solar energetic particles, SEP), and from galactic cosmic rays (GCR) also modifies material in the surface regolith. The intense ultraviolet radiation has very limited penetration depth. The solar wind has the highest fluence, but the low energy (∼1 keV nucleon^−1^) of the particles, chiefly protons, means that they are stopped in the outer few micrometers of surface materials (Table 1). While the solar wind is a constant feature of the lunar radiation environment, SEP occur only in occasional energetic outbursts from the Sun (Tripathi *et al.,* 2006; De Angelis *et al.,* 2007). SEP are dominated by protons, and energies generally range from 1 to 100 MeV per nucleon (Lucey *et al.,* 2006). Although the average fluence of SEP is much lower than the solar wind, the higher energies mean that SEP can penetrate the lunar regolith to depths of a few centimeters before being stopped. GCR are the highest-energy particles, consisting mainly of protons with a smaller component of heavy ions. The sources of GCR are thought mainly to be supernova explosions within the Galaxy (*e.g.*, Ackermann *et al.,* 2013) with some contribution from extragalactic sources, and particle energies range from around 100 MeV nucleon^−1^ up to tens of GeV nucleon^−1^. The extreme energies associated with GCR mean that particles striking planetary surfaces can penetrate several meters of rock and generate a cascade of highly damaging secondary particles within the regolith volume (*e.g.*, Dartnell, 2011).

**Table 1.** Summary of the Particle Radiation Types, Compositions, Energy, and Fluxes at the Lunar Surface

*Radiation*	*Particle types*	*Energy (MeV nucleon*^−1^*)*^a^	*Depth of penetration into regolith*	*Flux at lunar surface (protons cm*^−2^*s*^−1^*)*^b^
Solar wind	Protons (∼95%), alpha particles (∼4%), heavy ions (∼1%)^c^	∼0.001	A few μm	∼3 × 10^8^
Solar energetic particles	Protons (∼98%), alpha particles (∼2%), small fraction of heavy ions^a^	∼1–100	A few cm	∼0–10^6^
Galactic cosmic rays	Protons (∼87%), alpha particles (∼12%), heavy ions (∼1%)^a^	∼100–10,000	Several m	2–4

Data taken from ^a^Lucey *et al.* (2006), ^b^Vaniman *et al.* (1991), and ^c^Ogilvie and Coplan (1995).

**FIG. 1.**
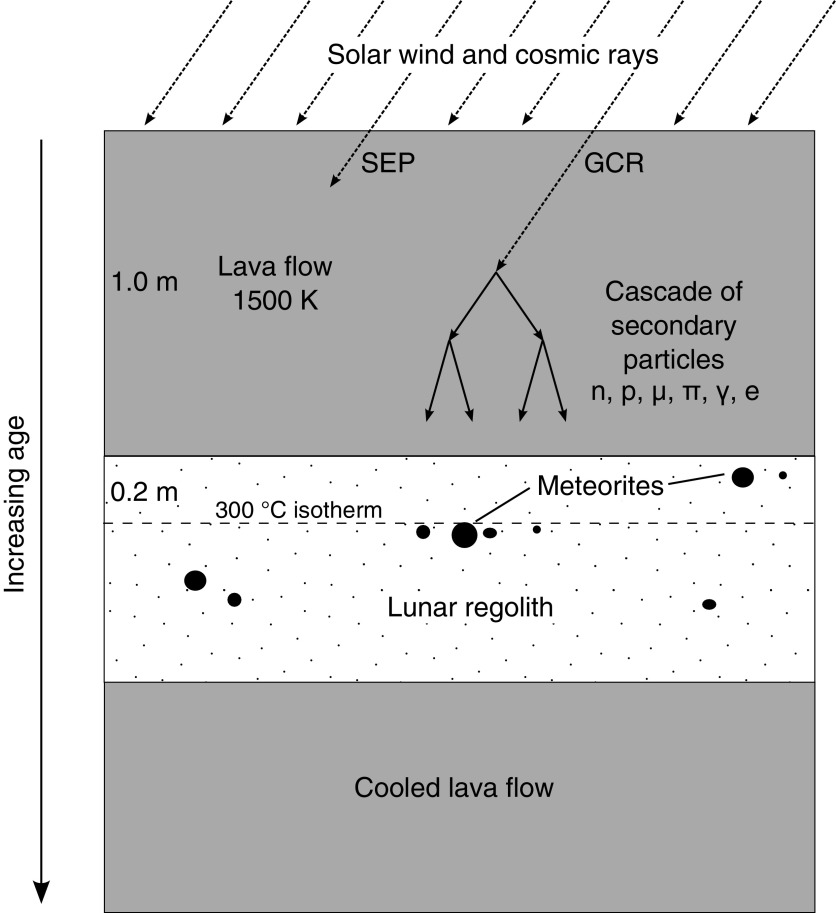
Schematic of the preservation of meteorites (black circles) within paleoregolith layers on the Moon. In this case, a 1.0 m thick lava flow has covered a thick layer of regolith containing meteorite fragments. The upper part of the regolith layer will be heated by the overlying lava, and the indicated depth of 0.2 m corresponds to a peak temperature of 300°C, which is reached after 20 days (Rumpf *et al.,* 2013). With a regolith accumulation rate of 5 mm Myr^−1^ (Hörz *et al.,* 1991), it would take 40 Myr for these meteorite fragments to be buried under 0.2 m of regolith. This assumes that there is no reworking to bring the meteorite to shallower depths in the regolith.

For any organic material to survive over geological periods of time and to be discoverable by an exploration mission, it must be protected from space weathering in some way. Ejecta blankets from nearby large impacts would be effective at quickly burying the material to substantial depth; however, it would be difficult to identify particular paleosurfaces within a sequence of very similar regolith layers. Rapid burial of preexisting regolith surfaces by pyroclastic deposits from fire-fountaining volcanic eruptions is another possibility (McKay, 2009), although such occurrences will be spatially and temporally limited. A mechanism of encapsulation by lava flows or impact melt flows provides the necessary combination of rapid burial and readily identifiable horizons within the paleoregolith layers (Crawford *et al.,* 2010; Fagents *et al.,* 2010; Rumpf *et al.,* 2013). In the lava flow preservation scenario, a fluid basaltic lava flow covers the surface regolith, which contains meteorites bearing the organic material of interest. The lava flow cools, and the solid basalt layer provides effective shielding for the buried regolith (Fig. 1). Space weathering processes then begin to form a regolith layer on the surface of the cooled basalt flow, incorporating any newly fallen meteoritic material. This new regolith layer is in turn covered by a new lava flow, and the process repeated. The result is a sequence of paleoregolith deposits and basalt lava layers. The individual basalt layers can be radiometrically dated, placing constraints on the duration of space exposure and closure age of the paleoregolith layers and the material within them. A record of the changes in the types of material being delivered to the lunar surface over time is thereby generated.

The lava flow preservation scenario has many advantages; however, the high temperatures of the lunar lava flows, with liquidus temperatures for mare basalts of between 1150°C and 1400°C (Taylor *et al.,* 1991), have the potential to alter and degrade organic materials contained within the underlying regolith layer. We have previously investigated the effect of heat from the overlying lava flow on different types of organic material that may be used to diagnose contributions from biological activity (*i.e.,* biomarkers), mixed with lunar regolith analog material. It was found that the presence of a regolith simulant promoted the preservation of intermixed organic matter (Matthewman *et al.,* 2015). Once buried beneath meters of cooled lava flows, the encapsulated organic matter would be protected from all but the most energetic GCR and associated particle cascades. However, before the lava flow can cover the regolith containing the carbonaceous particles, there is a period of time during which the organic matter will be subjected to radiation and micrometeorite impacts in the upper few centimeters (Fagents *et al.,* 2010; Rumpf *et al.,* 2013). Ionizing radiation is known to affect organic material in a number of ways (Swallow, 1960) and is damaging to biological materials. Common effects in organic matter include polymerization and cross-linking, with scission of long chains (Swallow, 1960; Chapiro, 1988). Experimental irradiation of meteoritic material caused progressive disordering and amorphization of the organic component (Brunetto *et al.,* 2014), and similar effects have been observed in the electron radiation of synthetic and terrestrial analog materials (Le Guillou *et al.,* 2013; Laurent *et al.,* 2014).

In this paper, we report an assessment of the effects of radiation on organic matter under conditions similar to those expected in lava-encapsulated records on the Moon. Our findings provide guidance for future lunar missions and their science goals.

## 2. Methods

To understand the effect that solar radiation might have on the preservation of abiotic and biotic organic compounds in the presence of lunar minerals, we subjected a range of organic materials to proton irradiation that was intended to simulate the effects of SEP.

### 2.1. Sample preparation

Table 2 lists the 12 samples that were irradiated, and Table 3 summarizes the organic materials used in the experiments. The organic materials are split into three categories: (1) hydrocarbon biomarkers, (2) polymer, and (3) amino acids:

(1) The hydrocarbon biomarkers used were hexadecane, squalene, squalane, coprostane, stigmasterol, anthracene, and pyrene. These hydrocarbons represent a variety of structural types of biotic and abiotic origin and have been used in previous extraterrestrially focused studies (Court *et al.,* 2010, 2012; Matthewman *et al.,* 2015).
(2) For the polymer, powdered (8 μm particle size) cross-linked poly(styrene-co-divinylbenzene) was used. It comprises long chains of styrene units, cross-linked by divinylbenzene. This polymer is intended to be representative of the aromatic macromolecular organic material in carbonaceous chondrite meteorites. Although structurally different, the polymer has been well characterized, and any structural or compositional modifications can be easily recognized.
(3) The amino acids used were glycine (Gly), l-alanine (Ala), l-aspartic acid (Asp), l-glutamic acid (Glu), and l-phenylalanine (Phe). Gly, Ala, Asp, and Glu are abundant in biological materials, whereas Phe, although present in biological materials, was primarily selected to represent amino acids containing an aromatic ring.

**Table 2.** Samples Subjected to Irradiation, Organic Material Used as Markers, and the Fluence Used

*Sample number*	*Organic material*	*Substrate*	*Total mass of organic material (mg)*	*Mass of substrate (mg)*	*Fluence (protons cm^−2^)*
1	Poly(styrene-co-divinylbenzene)	Quartz wool	3.74	—	2 × 10^14^
2		Quartz wool	2.83	—	3 × 10^13^
3		JSC-1	0.97	19.79	
4		MAC 88105 powder	0.99	18.85	
5		H_2_O/quartz wool	2.46	—	
6		Serpentinized mafic rock	1.03	19.62	
7	Hydrocarbon compounds	Quartz wool	∼0.07	—	3 × 10^13^
8		JSC-1	∼0.07	21.24	
9		MAC 88105 powder	∼0.07	20.00	
10	Amino acids	Quartz wool	∼0.05	—	3 × 10^13^
11		JSC-1	∼0.05	21.81	
12		MAC 88105 powder	∼0.05	19.95	

**Table 3.** Organic Materials Used in the Irradiation Experiments

*Hydrocarbon biomarkers*		*Amino acids*	
Hexadecane	C_16_H_34_	Glycine	C_2_H_5_NO_2_
Anthracene	C_14_H_10_	l-alanine	C_3_H_7_NO_2_
Pyrene	C_16_H_10_	l-aspartic acid	C_4_H_7_NO_4_
Squalane	C_30_H_62_	l-glutamic acid	C_5_H_9_NO_4_
Coprostane	C_27_H_48_	l-phenylalanine	C_9_H_11_NO_2_
Squalene	C_30_H_50_	*Polymeric materials*	
Stigmasterol	C_29_H_48_O	Poly(styrene-co-divinylbenzene)	

The organic materials were either used in isolation or were mixed with mineral substrates. To assess any effect of lunar regolith minerals on the organic materials during irradiation, organic samples were mixed with JSC-1, a lunar mare basaltic regolith analogue (for detailed characterization, see McKay *et al.,* 1994; Willman *et al.,* 1995), or with a powdered lunar feldspathic meteorite. The lunar meteorite used was MacAlpine Hills (MAC) 88105, subsplit 43 (0.203 g). This split was acquired in powder form from the Smithsonian Institute, where 20 g of the bulk parent meteorite stone had previously been processed to give a representative homogeneous powder (see Lindstrom *et al.,* 1991). This material was not modified or processed further before use in the experiments. MAC 88105 is a regolith breccia formed from consolidated feldspathic soil (Koeberl *et al.,* 1991; Lindstrom *et al.,* 1991; Neal *et al.,* 1991), which experienced low levels of space weathering [as indicated by a low I_s_/FeO space weathering index (Lindstrom *et al.,* 1995)]. The stone likely originated from the outer regions of the Feldspathic Highlands Terrane (FHT-O) (Joy *et al.,* 2010) and is, therefore, a good analogue of lunar highland soils.

Other minerals of relevance include those delivered in any carbonaceous meteorite, terrestrial meteorite, or cometary material itself. These minerals are likely to retain some degree of contact with organic material on the Moon during irradiation and other space weathering processes. As such, powdered serpentinized mafic rock was used as analog material for the meteorite mineral matrix. This mineral powder is loosely representative of both chondrites (Krot *et al.,* 2006) and aqueously altered terrestrial igneous rock that was likely to have been close to the surface of early Earth during the early Archean (*e.g.*, Kröner and Layer, 1992) and that may have contained organic materials, including some of biotic origin. The serpentinized rock contains a mix of olivine, pyroxene, and hydrated serpentine minerals. The JSC-1 powder was rinsed with dichloromethane to remove soluble organic contamination. The powdered serpentinized mafic rock was processed more extensively to remove soluble organic compounds by sonication in 93:7 dichloromethane/methanol v/v in triplicate. The MAC 88105 powder was used without cleaning with solvent.

For the free hydrocarbon compounds, a solution was made up in dichloromethane to a concentration of ∼1 mg mL^−1^ for each compound. Sample mineral powders were first placed inside quartz pyrolysis tubes and secured with quartz wool at the top and bottom. Ten microliters of the solution was spiked onto the substrate in the quartz sample tube and allowed to dry. For the amino acids, a solution was made up in high-purity 18 mΩ·cm water to a concentration of ∼2 mg mL^−1^ for each compound. Five microliters of this solution was spiked onto the substrate and allowed to dry. In all cases, the dry mass of mineral substrate was ∼20 mg. Where a mineral substrate was not used, solutions were spiked directly onto a loose plug of quartz wool.

The cross-linked poly(styrene-co-divinylbenzene) was thoroughly mixed with mineral powders to give a loading of ∼5 wt %. Approximately 20 mg of this mixture was used for each sample prepared for irradiation. Where a mineral matrix was not used, ∼2.5 to 3.8 mg of the polymer was placed directly into the quartz pyrolysis sample tube and secured with quartz wool. For sample 5 (Table 2), 18 mΩ·cm water was added to a sample tube containing poly(styrene-co-divinylbenzene).

The pyrolysis sample tubes were then flame sealed inside evacuated 6 mm (o.d.) borosilicate glass tubes. The exclusion of air was necessary to avoid reaction of the organic material with atmospheric oxygen during irradiation (Swallow, 1960). The samples containing water, free hydrocarbon compounds, and amino acid compounds were chilled in liquid nitrogen during vacuum flame sealing, to avoid the potential loss of compounds.

### 2.2. Irradiation

Samples in the sealed glass tubes were irradiated with protons at room temperature using the MC40 Scantronix cyclotron at the University of Birmingham. Table 2 shows the fluences used for each sample. The energy of the protons generated by the cyclotron was 25 MeV, which is within the energy spectrum of SEP (Table 1); however, irradiation of the samples was complicated by the glass tubes, which gave a variable target profile due to the cylindrical shape. Protons incident at the center of the tube had a shorter distance to travel through the glass than protons incident on the edge of the tube. The samples experienced a range of proton energies from ∼4 MeV (outside edge of the tube with maximum effective glass wall thickness) to ∼13 MeV (center of the tube with minimum effective glass wall thickness), calculated using SRIM-2013 with borosilicate glass as the model material. All but one sample was exposed to a fluence of 3 × 10^13^ protons cm^−2^ using a current of 800 nA and taking 13.2 min. For the single poly(styrene-co-divinylbenzene) sample exposed to the higher fluence, the current was 800 nA, and duration was ∼1.5 h, giving a fluence of 2 × 10^14^ protons cm^−2^. Confirmation that the samples were correctly aligned in the proton beam was provided by the use of radio-sensitive film placed behind the sample.

### 2.3. Sample recovery and solvent extraction

Following irradiation, the outer tubes of the samples containing the hydrocarbon biomarkers were wiped with acetone and cracked open. Clean steel wire was used to remove the sample from the inner pyrolysis tube. The inner tube was rinsed with dichloromethane/methanol 93:7 v/v and the solvent collected. The sample was sonicated in a test tube for 5 min in 0.5 mL dichloromethane/methanol 93:7 v/v, centrifuged, and the extract removed. The extraction was repeated for a total of three times, and the extracts were combined. The samples were filtered through quartz wool to remove residual mineral grains with dichloromethane rinses, then made up to 1 mL in dichloromethane ready for analysis.

To ascertain the recoveries of the hydrocarbon compounds from the substrates without the potential modifying effects of radiation, triplicate samples of quartz wool, MAC 88105, and JSC-1 substrates were spiked with the hydrocarbon mixture, vacuum sealed, and extracted, but were not irradiated.

The irradiated amino acids were extracted by using hot water. The outer sample tube was cracked open, and the small inner tube containing the sample was transferred to a new 6 mm o.d. borosilicate tube that had been sealed at one end. Two hundred microliters of 18 mΩ·cm water was added to the tube, before being chilled in liquid nitrogen and flame sealed under vacuum. These tubes were then heated at 100°C for 24 h. After heating, the outsides of the tubes were thoroughly cleaned with acetone and cracked open. Twenty-five microliters of the sample solution was transferred to a vial then dried at ∼35°C. Once completely dried, samples were derivatized by addition of 20 μL BSTFA and 10 μL pyridine, before being capped and heated at 85°C for 45 min immediately prior to analysis. Derivatization modifies the molecule into a form that is amenable to further analysis. Blank samples were also run to monitor any contamination introduced during the extraction and derivatization process.

### 2.4. Analysis

Hydrocarbon biomarkers were analyzed by gas chromatography–mass spectrometry (GC-MS). An Agilent 7890 GC and 5975C mass selective detector (MSD) system was used, with a 30 m J&W DB-5MS column with 250 μm i.d. and 0.25 μm film thickness. The GC oven was held at 50°C for 1 min then ramped at 4°C min^−1^ to 310°C, where it was held for 20 min. The helium flow rate was 1.1 mL min^−1^. The inlet was in splitless mode and held at a temperature of 250°C.

Polymer materials were analyzed with pyrolysis–gas chromatography–mass spectrometry (pyrolysis-GC-MS). Samples were analyzed by a Chemical Data Systems 5200 pyrolysis unit with an Agilent 7890 GC and 5975C MSD. The valve oven of the pyrolysis unit was maintained at 350°C and the transfer line at 270°C. The pyrolysis interface was rapidly ramped from 50°C at the start of the run to 350°C, taking less than a minute. Pyrolysis was performed at 600°C for 15 s. The pyrolysis products were separated on a 30 m J&W DB-5MS column with 250 μm i.d. and 0.25 μm film thickness. The oven was held at 40°C for 1 min before being ramped at 5°C min^−1^ to 310°C, where it was held for 10 min. The inlet was operated in split mode at a ratio of 50:1 and held at 270°C. The helium flow rate was 1.1 mL min^−1^.

Derivatized amino acid samples were analyzed with the Agilent 7890 GC and 5975C MSD with a 30 m J&W DB-5MS column. The GC oven was held at 80°C for 1 min, then heated at a rate of 8°C min^−1^ to 310°C, where it was held for 12 min. Helium flow rate was 1.1 mL min^−1^. The inlet was operated in splitless mode and held at 250°C.

The MSD scan range in all cases was from *m/z* 50 to 550. Compounds were identified by using retention times, comparison with standards, and the NIST08 mass spectrum library.

## 3. Results

Irradiation with protons under the experimental conditions described appeared to have had little effect on any of the compounds tested. No degradation products from any of the original organic materials were detected. The irradiation was sufficient to induce short-lived radioactivity in the sample tubes, as measured by a handheld detector, and caused brown discoloration of the outer glass tube.

### 3.1. Hydrocarbon biomarkers

**FIG. 2.**
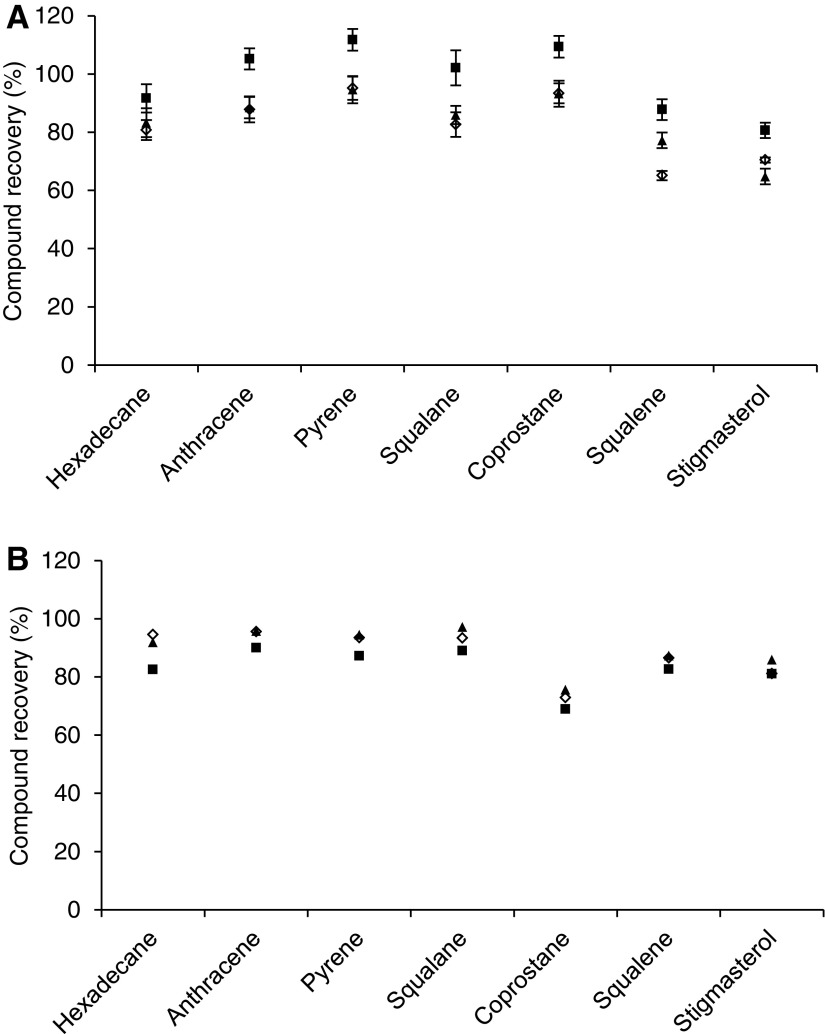
Recoveries of hydrocarbon compounds from (**A**) Non-irradiated samples, performed in triplicate. Error bars are 1 standard deviation. There is systematic variation between the different substrates, likely as a consequence of spiking with small volumes of volatile solvent, and (**B**) irradiated samples. The recoveries of the irradiated samples fall within the range of those from the non-irradiated samples. Open diamonds: quartz wool; triangles: JSC-1; squares: MAC 88105.

**FIG. 3.**
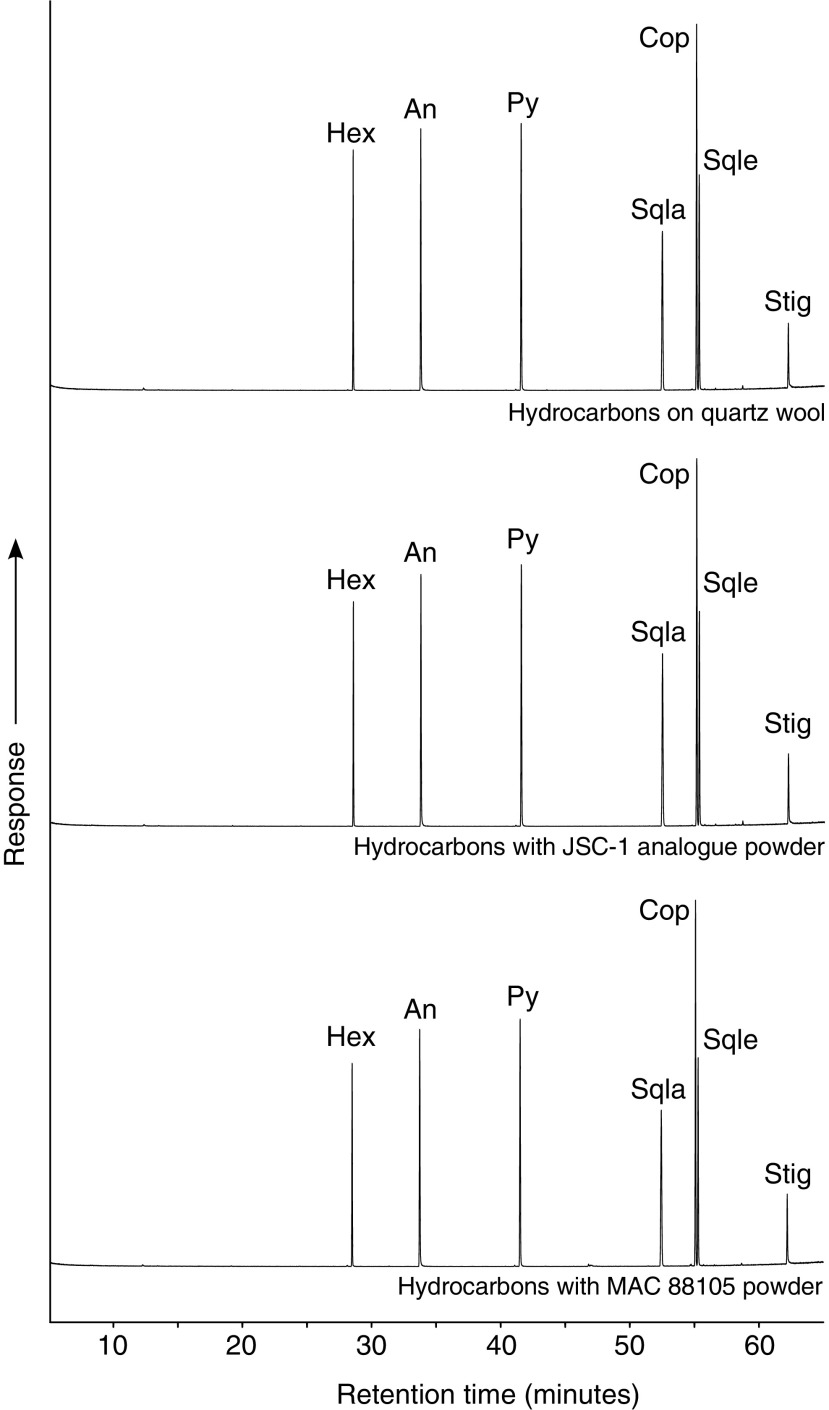
Total ion current chromatograms for the recovered hydrocarbons extracted from irradiated samples. Only the original compounds were detected, with no evidence for breakdown products above the limits of detection. Hex: hexadecane; An: anthracene; Py: pyrene; Sqla: squalane; Cop: coprostane; Sqle: squalene; Stig: stigmasterol.

The recovered fractions of the original hydrocarbon biomarker compounds following irradiation and the recoveries of hydrocarbon compounds that had not been subjected to irradiation are listed in Table 4 and plotted in Fig. 2. For the irradiated samples, it can be seen that the recoveries were high (>69%) across all three substrates. Notably, the recovery of coprostane was lower than the other compounds, by at least 14% compared with the recovery of hexadecane. This could be indicative of destruction by radiation; however, no degradation products were detected that would indicate breakdown of the molecule (Fig. 3). It would also require the irradiation to be selectively destructive. The stable rings of aromatic compounds are more resistant to the effects of ionizing radiation than aliphatic compounds (*e.g.*, LaVerne and Dowling-Medley, 2015, and references therein). However, the lack of molecular fragments from coprostane or other compounds requires an alternative explanation to the action of radiation which is responsible for the variation in compound recoveries. The recovery of hydrocarbon compounds from MAC 88105 is systematically lower than from the quartz wool and JSC-1 substrates (Table 4). The absence of compound fragments in the extract from MAC 88105 indicated that these losses were not the result of irradiation. One possible factor affecting recovery of the compounds could be adsorption onto the surfaces of glassware and mineral substrates. One of the primary controls on adsorption of organic materials to minerals is the size of the mineral grains. The MAC 88105 sample used was powdered to pass a 100-mesh sieve (Lindstrom *et al.,* 1991), which corresponds to a maximum grain size of 149 μm. Particles from JSC-1 have been measured up to several hundred micrometers across, with a median particle size of around 100 μm (McKay *et al.,* 1994; Willman *et al.,* 1995), indicating that a significant fraction of JSC-1 has a grain size greater than the maximum grain size of the powdered MAC 88105. The differences in grain sizes would result in higher overall surface area per unit mass for the powdered MAC 88105 than JSC-1, providing more opportunity for the adsorption of organic molecules and hence greater losses during extraction of MAC 88105. However, comparison with the recoveries from the non-irradiated samples showed that there was systematic variation that is more readily explained by errors from the handling of small volumes of volatile solutions, which may also be responsible for the lower recoveries of coprostane in the irradiated samples. Compounds structurally related to stigmasterol were detected in both extracted samples and in the standard solutions, indicating the presence of impurities in the original stigmasterol standard material. The recoveries of the experimentally irradiated compounds lie within the range of the non-irradiated recoveries, indicating that radiation has not had a substantially deleterious effect.

**Table 4.** Solvent Extraction Recoveries of Hydrocarbon Biomarker Compounds, Expressed as Percentage of the Original Mass

	*Compound recoveries (%)*								
	*Non-irradiated*						*Irradiated*		
	*Quartz wool*		*MAC 88105*		*JSC-1*				
	*Mean*	σ	*Mean*	σ	*Mean*	σ	*Quartz wool*	*MAC 88105*	*JSC-1*
Hexadecane	80.8	3.4	91.6	4.8	83.3	5.0	94.6	82.5	91.9
Anthracene	87.8	4.5	105.2	3.7	88.5	3.7	95.5	90.0	95.8
Pyrene	95.1	4.0	111.8	3.7	94.6	4.7	93.4	87.3	94.5
Squalane	82.6	4.2	102.1	6.0	85.9	3.2	93.4	89.0	97.1
Coprostane	93.4	3.4	109.4	3.7	93.3	4.5	72.9	68.9	75.6
Squalene	65.1	1.6	87.8	3.6	77.2	2.7	86.5	82.7	87.3
Stigmasterol	70.5	0.9	80.7	2.6	64.8	2.7	81.1	81.1	86.0

Non-irradiated samples were extracted to establish compound recoveries without the effects of radiation and were performed in triplicate to give standard deviations. Experimental limitations meant that only single examples of each substrate type could be irradiated.

### 3.2. Amino acids

**FIG. 4.**
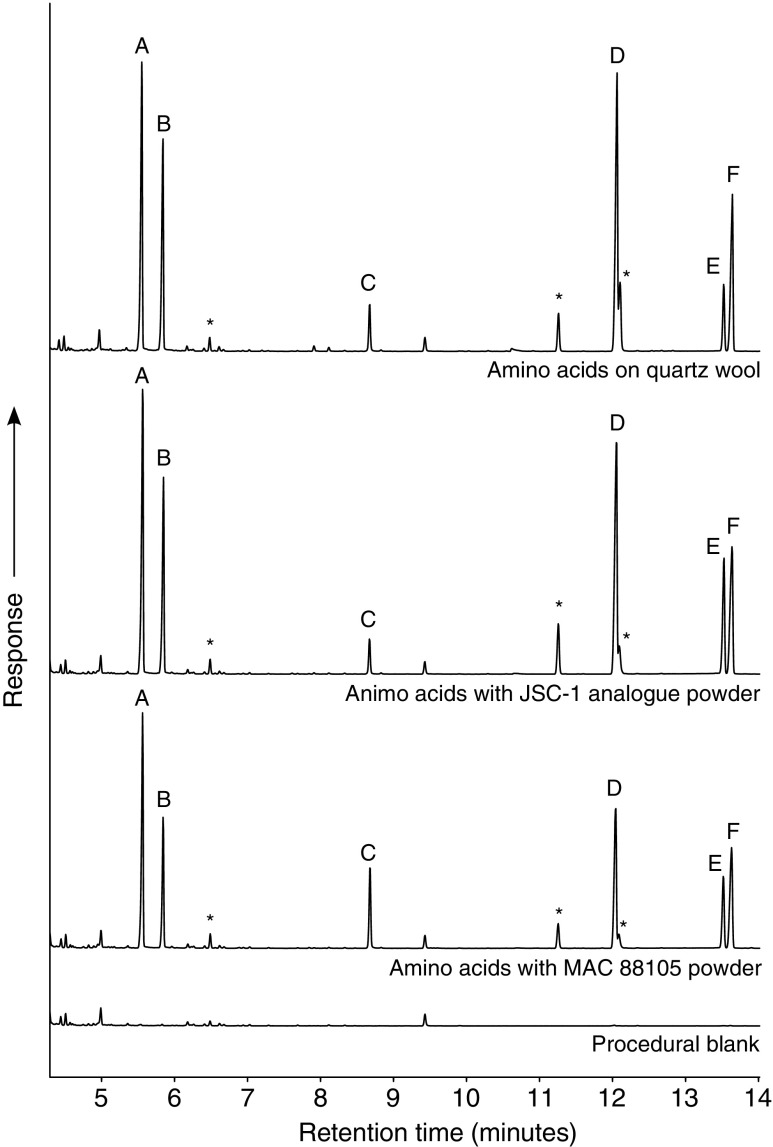
GC-MS total ion current chromatogram of the derivatized spiked amino acids irradiated by protons. Vertical scales are equivalent for each chromatogram. Peaks are derivatized versions of A: l-alanine; B, C: glycine; D: l-aspartic acid; E: l-glutamic acid; F: l-phenylalanine. A representative procedural blank is shown. The blank records any contamination introduced during the hot-water extraction process. Peaks labeled with an asterisk were also found in the chromatograms of non-irradiated amino acid standard mixtures and are therefore not products from the irradiation experiment.

Analysis of the amino acids was complicated by the requirement for derivatization to allow separation and detection by GC-MS. For some of the amino acids, it appeared that derivatization was incomplete, making quantification difficult. Nevertheless, the five amino acids were recovered from the irradiated samples in high concentrations, well above blank levels, and no products of degradation were detected. Figure 4 shows the chromatograms for the derivatized amino acids extracted from the irradiated samples. The relative responses of compounds between samples were similar, showing that the analog material did not have a substantially different effect to the genuine lunar material.

### 3.3. Polymers

None of the irradiated polymer samples showed any notable differences from non-irradiated polymer when using pyrolysis-GC-MS. The primary response in each sample was styrene (not shown). Styrene is a product from the breaking of the polymer chains during pyrolysis.

## 4. Discussion

Proton irradiation of a variety of organic compounds at fluences of 3 × 10^13^ protons cm^−2^ and 2 × 10^14^ protons cm^−2^ and energies between ∼4 and 13 MeV had little to no measurable effect on their recovery or composition. The presence or absence of different mineral powders mixed with the organic materials also had no apparent effect. The principal objective of the work was to determine if organic compounds were degraded or transformed into other products by proton radiation similar to SEP; no new compounds were detected, and there was no evidence for degradation in the form of molecular fragments of original compounds.

In the case of the high-molecular-weight polymer poly(styrene-co-divinylbenzene), no change to the units making up the polymer were detected. There have been many relevant studies of the irradiation of organic materials in space with application to the survival of polymers in spacecraft construction and instrument components (*e.g.*, Grossman and Gouzman, 2003). The main modes of alteration of a polymer in response to irradiation would be expected to be chain scission and cross-linking (Chapiro, 1995), but these effects were not observed in this study.

Recent work has demonstrated the presence of amino acids within lunar regolith samples returned from the Apollo missions (Elsila *et al.,* 2016). A portion of these amino acids were shown to be the result of terrestrial contamination; however, the source of other amino acids was less certain, and they could potentially be of meteoritic origin. Organic material associated with lunar pyroclastic deposits has also recently been described (Thomas-Keprta *et al.,* 2014). These results, together with the survival of organic compounds of multiple types in the irradiation experiments reported here, provide a renewed incentive to investigate curated regolith samples for the presence of organic materials by using sensitive modern equipment.

### 4.1. Radiation at the lunar surface

The radiation type simulated in this experimental work was SEP, but these represent only a fraction of the radiation types incident on the lunar surface. In addition, we were only able to simulate a limited part of the energy spectrum of SEP when using the monoenergetic cyclotron source (see Table 1). Ultraviolet radiation, the solar wind, and GCR including heavy ions were not simulated but may be important factors in the alteration and long-term preservation of organic matter on the surface of the Moon (*e.g.*, Sagan, 1972; Schwadron *et al.,* 2012).

Recent measurements from the Lunar Reconnaissance Orbiter have helped to refine our understanding of the lunar radiation environment (Spence *et al.,* 2010). The Cosmic Ray Telescope for the Effects of Radiation instrument indicates that dose deposition from GCR is sufficient to cause substantial space weathering effects (Schwadron *et al.,* 2012). Despite our improving knowledge of the present-day radiation environment, major uncertainties exist as to the flux of solar and cosmic radiation on the Moon over its lifetime. SEP are produced periodically by the Sun and can vary over long and short timescales (Vaniman *et al.,* 1991; Tripathi *et al.,* 2006). The averaged flux of SEP is believed to range between 50 and 100 protons cm^−2^ s^−1^ over the last 2–10 Myr (Reedy *et al.,* 1983; Rao *et al.,* 1994; Nishiizumi *et al.,* 2009). The equivalent ranges of duration of lunar exposure for the fluences used in our experiments are ∼9.5 to 19 kyr for the lower fluence and ∼63 to 127 kyr for the highest fluence. Our experimental durations are short in comparison to the length of time it would take to build up a layer of regolith of sufficient thickness to insulate against the heat from the overlying lava flow in the lava flow preservational model (Crawford *et al.,* 2010; Fagents *et al.,* 2010; Matthewman *et al.,* 2015). At a formation rate of 5 mm Myr^−1^, it would take 40 Myr to accumulate a regolith thickness of 0.2 m (Fig. 1). This rate is within the estimated range for the initial regolith formation rate at the Apollo 11 and 12 landing sites and can be compared with modern formation rates of ∼1 mm Myr^−1^ (Hörz *et al.,* 1991). Our work has not identified a maximum duration on the lunar surface following which organic preservation is retained. Yet the lack of degradation in our experiments can be interpreted conservatively to indicate that in those circumstances where regolith is deposited relatively quickly there appears to be no barrier to effective preservation. We note that total radiolytic degradation of organic compounds increases by several orders of magnitude over timescales of millions of years (Kminek and Bada, 2006) and that SEP are variable over time (Vaniman *et al.,* 1991; Tripathi *et al.,* 2006), and further complex experiments will be required for an exhaustive assessment.

Beneath the maximum depth of penetration of the most energetic cosmic rays, the primary sources of radiation on the Moon would be mineral grains and glass phases containing radioactive elements (Lucey *et al.,* 2006). The influence of radiation from radioactive mineral sources would depend on the dominant rock type. For example, KREEP-rich samples (enriched in potassium, phosphorous, and rare earth elements) like KREEP basalts, KREEP-rich impact melts, and High Alkali Suite rocks, have relatively high concentrations of the incompatible elements uranium and thorium (Vaniman *et al.,* 1991; Korotev, 1998; Yamashita *et al.,* 2010). Radioactive components in these rocks could modify exogenous organic material that had become incorporated. However, the range of influence of alpha radiation from these grains is limited to tens of micrometers (Owen, 1988; Nasdala *et al.,* 2006). Short-lived radioactive nuclides that persisted from the formation of the Solar System (Chaussidon and Gounelle, 2007) may have provided an additional source of radiation that is absent today. Mineral radioactivity induces polymerization of smaller molecules and changes the structure of aromatic organic materials (Court *et al.,* 2006, 2007).

The flux of radiation at the lunar surface and within the regolith is likely to have varied over the lifetime of the Moon. The flux of the solar wind during the early history of the Sun is believed to have been hundreds of times higher than today (Wood *et al.,* 2005; Cnossen *et al.,* 2007; Lundin *et al.,* 2007). It would therefore be expected that degradation of organic matter due to solar particle radiation would be higher at this time.

A further consideration is the length of time that a terrestrial meteorite spends in transit from Earth to the Moon after being ejected by impact (*i.e.,* 4π irradiation). Transit times for asteroidal meteorites to Earth commonly vary from a few million years to hundreds of millions of years (Eugster *et al.,* 2006), and the meteorites will be exposed to space radiation for the duration. However, the transfer time for a terrestrial meteorite to the Moon is likely to be less than a few thousand years (Armstrong *et al.,* 2002), which is a short time relative to the potential residence time of the meteorite once it has landed on the lunar surface.

### 4.2. Regolith formation processes and preservation of meteorites

The discovery of meteoritic organic compounds with a recognizable source in layered paleoregolith deposits on the Moon will most likely involve centimeter-scale meteorites that have escaped substantial comminution. Meteorites of this size would have sufficient mineralogical information to help constrain whether they originated from an asteroid or cometary or planetary body (Joy *et al.,* 2012). The ultraviolet radiation and the high flux of low-energy solar wind particles together with micrometeorite impacts will destroy or heavily degrade organic compounds in the outer layers of a meteorite at the surface of the lunar regolith. However, in larger meteorite fragments, the internal portion of the meteorite will be shielded from these types of radiation, which only penetrate to shallow depths in rock. Only the more energetic GCR and SEP, along with radiation from cascades, will affect the compounds preserved in the center of these meteorites.

A preservation scenario that minimizes the duration of exposure of the organic meteoritic material to radiation would reduce the opportunity for degradation. Our experiments simulated exposure to SEP for tens of thousands of years. However, in a “steady state”–type model, the slow and gradual formation of regolith of sufficient thickness to bury and protect meteoritic material would take on the order of tens of millions of years (Fig. 1). Yet the lunar surface early in the history of the Earth-Moon system was a chaotic place, with a higher frequency of impacts than occurs today. As a result, regolith formation rates may have been substantially higher than today (Fagents *et al.,* 2010). Even small impacts would have generated ejecta blankets thick enough to protect underlying materials (*i.e.*, megaregolith formation), and the seismic shocks caused by impact would have caused slope failures and landslides (Schultz and Gault, 1975; Xiao *et al.,* 2013); these events could have quickly buried meteorites lying in and on surficial regolith. Pyroclastic deposits from fire-fountaining volcanic eruptions would have a similar effect (McKay, 2009). A number of scenarios can, therefore, be envisioned for the creation of meteorite lagerstätten in lunar regolith, reducing the length of exposure of exogenous organic materials to micrometeorite impacts and radiation. However, identifying these horizons and determining their closure ages may be more challenging than for the paleoregolith lava flow preservation scenario. Correlation of known increases in impactor flux through geological time on Earth with dated layers on the Moon would help identify paleoregolith layers that potentially contain high concentrations of meteorites (*e.g.*, Schmitz *et al.,* 2001; Jones, 2014).

The quantity of organic material within an asteroidal or terrestrial meteorite that is delivered to the surface of the Moon will also affect how likely it is that detectable quantities of organic compounds are preserved. For a given rate of degradation, meteorites with a high concentration of organic compounds would be more likely to retain some organic chemical information following irradiation compared to an organic-poor meteorite. Carbonaceous asteroidal meteorites contain a range of proportions of organic material, depending on their type and alteration history. The CM2 chondrite Murchison is perhaps the most extensively analyzed example of an organic-rich asteroidal meteorite. The types and abundances of organic materials within Murchison have been summarized by Sephton (2002). The majority of the organic matter is insoluble macromolecular material (1.45%), which is represented by the ∼5 wt % of poly(styrene-co-divinylbenzene) added to the mineral substrates in this study. Soluble hydrocarbons, amino acids, and carboxylic acids in the Murchison meteorite are each in the range of tens to hundreds of parts per million. The meteorite soluble organic compound abundances are lower than those for the spiked substrates used in this study (∼0.35 wt % for hydrocarbons and ∼0.25 wt % for amino acids).

The concentration of organic material in terrestrial meteorites that may have been delivered to the surface of the Moon billions of years ago is uncertain. The rock record from this time has largely been destroyed, and indeed it is perhaps terrestrial meteorites preserved on the Moon that will provide the best constraints on the organic chemical and biological conditions on the earliest Earth (Armstrong *et al.,* 2002; Armstrong, 2010). Hydrothermal vent systems have been suggested as possible sites for early life to thrive (*e.g.*, Martin *et al.,* 2008), and modern carbonate samples from the Atlantic Lost City hydrothermal field show total organic carbon values of up to 0.6% (Kelley *et al.,* 2005). Such modern ecosystems that can act as analogues for unpreserved ancient counterparts provide an indication of the possible abundances of organic matter that could have been transferred to the Moon.

The measured abundances of organic material in asteroidal meteorites and analog biotic sites, and the types of organic compound within them, provide starting points for estimating what fraction of material may remain after encapsulation for billions of years in lunar paleoregolith layers. The experimental radiation of hydrocarbons, amino acids, and polymers has shown that recoveries of the original compounds remain high after proton irradiation and that no secondary breakdown products were detected. This would suggest that the degradation of organic material at the lunar surface owing to radiation may occur at slow rates, and as such a high proportion of the original meteoritic organic material would remain unaffected. In the case where radiation has a significant deleterious effect on organic matter, it would be expected that polycyclic aromatic hydrocarbon–dominated structures would be the most persistent types of organic material (Court *et al.,* 2006).

### 4.3. Irradiation of organic materials in the presence of water

To investigate the influence of aqueous fluids, one of our polymer samples was irradiated while immersed in water. Our data are relevant to potential organic carbon and water-ice mixtures that may occur in permanently shadowed craters at the poles of the Moon (Colaprete *et al.,* 2010; Crites *et al.,* 2013). Organic molecules can be generated and modified by interaction with ices under irradiation (Bernstein *et al.,* 1995; Lucey, 2000; Hand and Carlson, 2012; Crites *et al.,* 2013). Although the permanently shadowed regions are largely protected from the effects of ultraviolet radiation, they are still subject to GCR (Schwadron *et al.,* 2012) and the solar wind (Zimmerman *et al.,* 2011). Irradiation of water and water ice can result in the formation of hydrogen and hydroxyl radicals, which can also recombine to form hydrogen peroxide (Johnson and Quickenden, 1997; Loeffler *et al.,* 2006; Dartnell, 2011). These highly reactive species have the potential to degrade organic matter. However, no evidence for degradation of the organic materials by irradiation in the presence of water was found in our data.

## 5. Conclusion

Proton irradiation intended to simulate exposure of representative biotic and abiotic polymers and compounds to SEP at the lunar surface over periods of tens of thousands of years had little to no effect on the organic materials under the experimental conditions used. The presence or absence of lunar meteorite powder and other mineral analogues mixed with the organic materials had no apparent influence. The data demonstrate the relatively robust nature of the organic materials and suggests that they have a high preservation potential in the lunar radiation environment if buried and encapsulated on favorable timescales. However, the proton energy used in the experiments of ∼4–13 MeV at the sample is representative of only the lower energy range of SEP. SEP in turn is only one type of radiation that occurs on the lunar surface environment. Lower-energy but higher-fluence radiation types may have an influence on the preservation of organic matter where reworking and micrometeorite impacts in the regolith may expose the organic molecules to the space environment.

The relative resistance of a range of organic materials to the influence of radiation promotes optimism for the detection and interpretation of *in situ* and *ex situ* organic assemblages on the Moon. In particular, encapsulated organic records of impact-ejected planetary surfaces on the Moon may not be extensively corrupted by the radiation environment. Our results, therefore, suggest that prebiotic and biotic chemistry from early Earth, and possibly from elsewhere in the Solar System, could be preserved in the Moon's near-surface geological record. Identifying such materials will be an important objective for future lunar exploration missions.

## Abbreviations Used

GC-MS: gas chromatography–mass spectrometry
GCR: galactic cosmic rays
LHB: Late Heavy Bombardment
MAC: MacAlpine Hills
MSD: mass selective detector
pyrolysis-GC-MS: pyrolysis–gas chromatography–mass spectrometry
SEP: solar energetic particles

## References

[B1] Ackermann M., Ajello M., Allafort A., Baldini L., Ballet J., Barbiellini G., Baring M.G., Bastieri D., Bechtol K., Bellazzini R., Blandford R.D., Bloom E.D., Bonamente E., Borgland A.W., Bottacini E., Brandt T.J., Bregeon J., Brigida M., Bruel P., Buehler R., Busetto G., Buson S., Caliandro G.A., Cameron R.A., Caraveo P.A., Casandjian J.M., Cecchi C., Çelik Ö., Charles E., Chaty S., Chaves R.C.G., Chekhtman A., Cheung C.C., Chiang J., Chiaro G., Cillis A.N., Ciprini S., Claus R., Cohen-Tanugi J., Cominsky L.R., Conrad J., Corbel S., Cutini S., D'Ammando F., de Angelis A., de Palma F., Dermer C.D., do Couto e Silva E., Drell P.S., Drlica-Wagner A., Falletti L., Favuzzi C., Ferrara E.C., Franckowiak A., Fukazawa Y., Funk S., Fusco P., Gargano F., Germani S., Giglietto N., Giommi P., Giordano F., Giroletti M., Glanzman T., Godfrey G., Grenier I.A., Grondin M.-H., Grove J.E., Guiriec S., Hadasch D., Hanabata Y., Harding A.K., Hayashida M., Hayashi K., Hays E., Hewitt J.W., Hill A.B., Hughes R.E., Jackson M.S., Jogler T., Jóhannesson G., Johnson A.S., Kamae T., Kataoka J., Katsuta J., Knödlseder J., Kuss M., Lande J., Larsson S., Latronico L., Lemoine-Goumard M., Longo F., Loparco F., Lovellette M.N., Lubrano P., Madejski G.M., Massaro F., Mayer M., Mazziotta M.N., McEnery J.E., Mehault J., Michelson P.F., Mignani R.P., Mitthumsiri W., Mizuno T., Moiseev A.A., Monzani M.E., Morselli A., Moskalenko I.V., Murgia S., Nakamori T., Nemmen R., Nuss E., Ohno M., Ohsugi T., Omodei N., Orienti M., Orlando E., Ormes J.F., Paneque D., Perkins J.S., Pesce-Rollins M., Piron F., Pivato G., Rainò S., Rando R., Razzano M., Razzaque S., Reimer A., Reimer O., Ritz S., Romoli C., Sánchez-Conde M., Schulz A., Sgrò C., Simeon P.E., Siskind E.J., Smith D.A., Spandre G., Spinelli P., Stecker F.W., Strong A.W., Suson D.J., Tajima H., Takahashi H., Takahashi T., Tanaka T., Thayer J.G., Thayer J.B., Thompson D.J., Thorsett S.E., Tibaldo L., Tibolla O., Tinivella M., Troja E., Uchiyama Y., Usher T.L., Vandenbroucke J., Vasileiou V., Vianello G., Vitale V., Waite A.P., Werner M., Winer B.L., Wood K.S., Wood M., Yamazaki R., Yang Z., and Zimmer S. (2013) Detection of the characteristic pion-decay signature in supernova remnants. Science 339:807–811 23413352 10.1126/science.1231160

[B2] Anand M. (2010) Lunar water: a brief review. Earth Moon Planets 107:65–73

[B3] Armstrong J.C. (2010) Distribution of impact locations and velocities of Earth meteorites on the Moon. Earth Moon Planets 107:43–54

[B4] Armstrong J.C., Wells L.E., and Gonzalez G. (2002) Rummaging through Earth's attic for remains of ancient life. Icarus 160:183–196

[B5] Bernstein M.P., Sandford S.A., Allamandola L.J., Chang S., and Scharberg M.A. (1995) Organic compounds produced by photolysis of realistic interstellar and cometary ice analogs containing methanol. Astrophys J 454:327–344

[B6] Bottke W.F., Vokrouhlicky D., Minton D., Nesvorny D., Morbidelli A., Brasser R., Simonson B., and Levison H.F. (2012) An Archaean heavy bombardment from a destabilized extension of the asteroid belt. Nature 485:78–81 22535245 10.1038/nature10967

[B7] Brunetto R., Lantz C., Ledu D., Baklouti D., Barucci M.A., Beck P., Delauche L., Dionnet Z., Dumas P., Duprat J., Engrand C., Jamme F., Oudayer P., Quirico E., Sandt C., and Dartois E. (2014) Ion irradiation of Allende meteorite probed by visible, IR, and Raman spectroscopies. Icarus 237:278–292

[B8] Burchell M.J., McDermott K.H., Price M.C., and Yolland L.J. (2014) Survival of fossils under extreme shocks induced by hypervelocity impacts. Philos Trans A Math Phys Eng Sci 372, doi:10.1098/rsta.2013.0190 PMC4115461 25071234

[B9] Capaccioni F., Coradini A., Filacchione G., Erard S., Arnold G., Drossart P., De Sanctis M.C., Bockelee-Morvan D., Capria M.T., Tosi F., Leyrat C., Schmitt B., Quirico E., Cerroni P., Mennella V., Raponi A., Ciarniello M., McCord T., Moroz L., Palomba E., Ammannito E., Barucci M.A., Bellucci G., Benkhoff J., Bibring J.P., Blanco A., Blecka M., Carlson R., Carsenty U., Colangeli L., Combes M., Combi M., Crovisier J., Encrenaz T., Federico C., Fink U., Fonti S., Ip W.H., Irwin P., Jaumann R., Kuehrt E., Langevin Y., Magni G., Mottola S., Orofino V., Palumbo P., Piccioni G., Schade U., Taylor F., Tiphene D., Tozzi G.P., Beck P., Biver N., Bonal L., Combe J.-P., Despan D., Flamini E., Fornasier S., Frigeri A., Grassi D., Gudipati M., Longobardo A., Markus K., Merlin F., Orosei R., Rinaldi G., Stephan K., Cartacci M., Cicchetti A., Giuppi S., Hello Y., Henry F., Jacquinod S., Noschese R., Peter G., Politi R., Reess J.M., and Semery A. (2015) The organic-rich surface of comet 67P/Churyumov-Gerasimenko as seen by VIRTIS/Rosetta. Science 347, doi:10.1126/science.aaa0628 25613895

[B10] Chapiro A. (1988) Chemical modifications in irradiated polymers. Nucl Instrum Methods Phys Res B 32:111–114

[B11] Chapiro A. (1995) General consideration of the radiation chemistry of polymers. Nucl Instrum Methods Phys Res B 105:5–7

[B12] Chaussidon M. and Gounelle M. (2007) Short-lived radioactive nuclides in meteorites and early solar system processes. Comptes Rendus Geoscience 339:872–884

[B13] Chyba C. and Sagan C. (1992) Endogenous production, exogenous delivery and impact-shock synthesis of organic molecules: an inventory for the origins of life. Nature 355:125–132 11538392 10.1038/355125a0

[B14] Cnossen I., Sanz-Forcada J., Favata F., Witasse O., Zegers T., and Arnold N.F. (2007) Habitat of early life: solar X-ray and UV radiation at Earth's surface 4–3.5 billion years ago. J Geophys Res Planets 112, doi:10.1029/2006JE002784

[B15] Colaprete A., Schultz P., Heldmann J., Wooden D., Shirley M., Ennico K., Hermalyn B., Marshall W., Ricco A., Elphic R.C., Goldstein D., Summy D., Bart G.D., Asphaug E., Korycansky D., Landis D., and Sollitt L. (2010) Detection of water in the LCROSS ejecta plume. Science 330:463–468 20966242 10.1126/science.1186986

[B16] Court R.W. and Sephton M.A. (2014) New estimates of the production of volatile gases from ablating carbonaceous micrometeoroids at Earth and Mars during an E-belt-type Late Heavy Bombardment. Geochim Cosmochim Acta 145:175–205

[B17] Court R.W., Sephton M.A., Parnell J., and Gilmour I. (2006) The alteration of organic matter in response to ionising irradiation: chemical trends and implications for extraterrestrial sample analysis. Geochim Cosmochim Acta 70:1020–1039

[B18] Court R.W., Sephton M.A., Parnell J., and Gilmour I. (2007) Raman spectroscopy of irradiated organic matter. Geochim Cosmochim Acta 71:2547–2568

[B19] Court R.W., Baki A.O., Sims M.R., Cullen D., and Sephton M.A. (2010) Novel solvent systems for *in situ* extraterrestrial sample analysis. Planet Space Sci 58:1470–1474

[B20] Court R.W., Rix C.S., Sims M.R., Cullen D.C., and Sephton M.A. (2012) Extraction of polar and nonpolar biomarkers from the martian soil using aqueous surfactant solutions. Planet Space Sci 67:109–118

[B21] Crawford I.A., Baldwin E.C., Taylor E.A., Bailey J.A., and Tsembelis K. (2008) On the survivability and detectability of terrestrial meteorites on the Moon. Astrobiology 8:242–252 18393690 10.1089/ast.2007.0215

[B22] Crawford I.A., Fagents S.A., Joy K.H., and Rumpf M.E. (2010) Lunar palaeoregolith deposits as recorders of the galactic environment of the Solar System and implications for astrobiology. Earth Moon Planets 107:75–85

[B23] Crites S.T., Lucey P.G., and Lawrence D.J. (2013) Proton flux and radiation dose from galactic cosmic rays in the lunar regolith and implications for organic synthesis at the poles of the Moon and Mercury. Icarus 226:1192–1200

[B24] Dartnell L.R. (2011) Ionizing radiation and life. Astrobiology 11:551–582 21774684 10.1089/ast.2010.0528

[B25] De Angelis G., Badavi F.F., Clem J.M., Blattnig S.R., Clowdsley M.S., Nealy J.E., Tripathi R.K., and Wilson J.W. (2007) Modeling of the lunar radiation environment. Nucl Phys B Proc Suppl 166:169–183

[B26] Ehrenfreund P. and Charnley S.B. (2000) Organic molecules in the interstellar medium, comets, and meteorites: a voyage from dark clouds to the early Earth. Annu Rev Astron Astrophys 38:427–483

[B27] Elsila J.E., Callahan M.P., Dworkin J.P., Glavin D.P., McLain H.L., Noble S.K., and Gibson E.K. Jr. (2016) The origin of amino acids in lunar regolith samples. Geochim Cosmochim Acta 172:357–369

[B28] Eugster O., Herzog G.F., Marti K., and Caffee M.W. (2006) Irradiation records, cosmic-ray exposure ages, and transfer times of meteorites. In Meteorites and the Early Solar System II, edited by Lauretta D.S. and McSween H.Y., The University of Arizona Press, Tucson, AZ, pp 829–851

[B29] Fagents S.A., Rumpf M.E., Crawford I.A., and Joy K.H. (2010) Preservation potential of implanted solar wind volatiles in lunar paleoregolith deposits buried by lava flows. Icarus 207:595–604

[B30] Gomes R., Levison H.F., Tsiganis K., and Morbidelli A. (2005) Origin of the cataclysmic Late Heavy Bombardment period of the terrestrial planets. Nature 435:466–469 15917802 10.1038/nature03676

[B31] Grossman E. and Gouzman I. (2003) Space environment effects on polymers in low Earth orbit. Nucl Instrum Methods Phys Res B 208:48–57

[B32] Hand K.P. and Carlson R.W. (2012) Laboratory spectroscopic analyses of electron irradiated alkanes and alkenes in Solar System ices. J Geophys Res Planets 117, doi:10.1029/2011JE003888

[B33] Hörz F., Grieve R., Heiken G., Spudis P., and Binder A. (1991) Lunar surface processes. In The Lunar Sourcebook: A User's Guide to the Moon, edited by Heiken G.H., Vaniman D.T., and French B.M., Cambridge University Press, Cambridge, UK, pp 61–120

[B34] Johnson R.E. and Quickenden T.I. (1997) Photolysis and radiolysis of water ice on outer Solar System bodies. J Geophys Res Planets 102:10985–10996

[B35] Jones A.P. (2014) Impact volcanism and mass extinctions. GSA Special Papers 505:369–381

[B36] Joy K.H., Crawford I.A., Russell S.S., and Kearsley A.T. (2010) Lunar meteorite regolith breccias: an *in situ* study of impact melt composition using LA-ICP-MS with implications for the composition of the lunar crust. Meteorit Planet Sci 45:917–946

[B37] Joy K.H., Zolensky M.E., Nagashima K., Huss G.R., Ross D.K., McKay D.S., and Kring D.A. (2012) Direct detection of projectile relics from the end of the lunar basin–forming epoch. Science 336:1426–1429 22604725 10.1126/science.1219633

[B38] Keller L.P. and McKay D.S. (1997) The nature and origin of rims on lunar soil grains. Geochim Cosmochim Acta 61:2331–2341

[B39] Kelley D.S., Karson J.A., Früh-Green G.L., Yoerger D.R., Shank T.M., Butterfield D.A., Hayes J.M., Schrenk M.O., Olson E.J., Proskurowski G., Jakuba M., Bradley A., Larson B., Ludwig K., Glickson D., Buckman K., Bradley A.S., Brazelton W.J., Roe K., Elend M.J., Delacour A., Bernasconi S.M., Lilley M.D., Baross J.A., Summons R.E., and Sylva S.P. (2005) A serpentinite-hosted ecosystem: the Lost City hydrothermal field. Science 307:1428–1434 15746419 10.1126/science.1102556

[B40] Kminek G. and Bada J.L. (2006) The effect of ionizing radiation on the preservation of amino acids on Mars. Earth Planet Sci Lett 245:1–5

[B41] Koeberl C., Kurat G., and Brandstätter F. (1991) MAC88105—a regolith breccia from the lunar highlands: mineralogical, petrological, and geochemical studies. Geochim Cosmochim Acta 55:3073–3087

[B42] Korotev R.L. (1998) Concentrations of radioactive elements in lunar materials. J Geophys Res: Planets 103:1691–1701

[B43] Kring D.A. and Cohen B.A. (2002) Cataclysmic bombardment throughout the inner Solar System 3.9–4.0 Ga. J Geophys Res: Planets 107, doi:10.1029/2001JE001529

[B44] Kröner A. and Layer P.W. (1992) Crust formation and plate motion in the Early Archean. Science 256:1405–1411 17791608 10.1126/science.256.5062.1405

[B45] Krot A.N., Hutcheon I.D., Brearley A.J., Pravdivtseva M.I., Petaev C.M., and Hohenberg C.M. (2006) Timescales and settings for alteration of chondritic meteorites. In Meteorites and the Early Solar System II, edited by Lauretta D.S. and McSween H.Y., The University of Arizona Press, Tucson, AZ, pp 525–553

[B46] Laurent B., Roskosz M., Remusat L., Leroux H., Vezin H., and Depecker C. (2014) Isotopic and structural signature of experimentally irradiated organic matter. Geochim Cosmochim Acta 142:522–534

[B47] LaVerne J.A. and Dowling-Medley J. (2015) Combinations of aromatic and aliphatic radiolysis. J Phys Chem A 119:10125–10129 26387844 10.1021/acs.jpca.5b07414

[B48] Le Guillou C., Remusat L., Bernard S., Brearley A.J., and Leroux H. (2013) Amorphization and D/H fractionation of kerogens during experimental electron irradiation: comparison with chondritic organic matter. Icarus 226:101–110

[B49] Levison H.F., Dones L., Chapman C.R., Stern S.A., Duncan M.J., and Zahnle K. (2001) Could the lunar “Late Heavy Bombardment” have been triggered by the formation of Uranus and Neptune? Icarus 151:286–306

[B50] Lindstrom M.M., Schwarz C., Score R., and Masons B. (1991) MacAlpine Hills 88104 and 88105 lunar highland meteorites: general description and consortium overview. Geochim Cosmochim Acta 55:2999–3007

[B51] Lindstrom M.M., Mittlefehldt D.W., Morris R.V., Martinez R.R., and Wentworth S.J. (1995) QUE93069, a more mature regolith breccia for the Apollo 25^th^ anniversary. In 26^th^ Lunar and Planetary Science Conference Abstracts, Lunar and Planetary Institute, Houston, pp 849–850

[B52] Loeffler M.J., Raut U., Vidal R.A., Baragiola R.A., and Carlson R.W. (2006) Synthesis of hydrogen peroxide in water ice by ion irradiation. Icarus 180:265–273

[B53] Love S.G. and Brownlee D.E. (1993) A direct measurement of the terrestrial mass accretion rate of cosmic dust. Science 262:550–553 17733236 10.1126/science.262.5133.550

[B54] Lucey P., Korotev R.L., Gillis J.J., Taylor L.A., Lawrence D., Campbell B.A., Elphic R., Feldman B., Hood L.L., Hunten D., Mendillo M., Noble S., Papike J.J., Reedy R.C., Lawson S., Prettyman T., Gasnault O., and Maurice S. (2006) Understanding the lunar surface and space-Moon interactions. Reviews in Mineralogy and Geochemistry 60:83–219

[B55] Lucey P.G. (2000) Potential for prebiotic chemistry at the poles of the Moon. Proc SPIE 4137, doi:10.1117/12.411612

[B56] Lundin R., Lammer H., and Ribas I. (2007) Planetary magnetic fields and solar forcing: implications for atmospheric evolution. Space Sci Rev 129:245–278

[B57] Marchi S., Bottke W.F., Elkins-Tanton L.T., Bierhaus M., Wuennemann K., Morbidelli A., and Kring D.A. (2014) Widespread mixing and burial of Earth's Hadean crust by asteroid impacts. Nature 511:578–582 25079556 10.1038/nature13539

[B58] Martin W., Baross J., Kelley D., and Russell M.J. (2008) Hydrothermal vents and the origin of life. Nat Rev Microbiol 6:805–814 18820700 10.1038/nrmicro1991

[B59] Matthewman R., Court R.W., Crawford I.A., Jones A.P., Joy K.H., and Sephton M.A. (2015) The Moon as a recorder of organic evolution in the early Solar System: a lunar regolith analog study. Astrobiology 15:154–168 25615648 10.1089/ast.2014.1217 PMC4322787

[B60] McDermott J.M., Seewald J.S., German C.R., and Sylva S.P. (2015) Pathways for abiotic organic synthesis at submarine hydrothermal fields. Proc Natl Acad Sci USA 112:7668–7672 26056279 10.1073/pnas.1506295112 PMC4485091

[B61] McKay D.S. (2009) Do lunar pyroclastic deposits contain the secrets of the Solar System? In Lunar Reconnaissance Orbiter Science Targeting Meeting, LPI Contribution No. 1483, Lunar and Planetary Institute, Houston, pp 77–78

[B62] McKay D.S., Carter J.L., Boles W.W., Allen C.C., and Allton J.H. (1994) JSC-1: a new lunar soil simulant. In Engineering, Construction, and Operations in Space IV: Proceedings of Space 94, edited by Galloway R.G. and Lokaj S., American Society of Civil Engineers, New York, pp 857–866

[B63] McSween H.Y. (1976) A new type of chondritic meteorite found in lunar soil. Earth Planet Sci Lett 31:193–199

[B64] Nasdala L., Wildner M., Wirth R., Groschopf N., Pal D.C., and Möller A. (2006) Alpha particle haloes in chlorite and cordierite. Mineralogy and Petrology 86:1–27

[B65] Neal C.R., Taylor L.A., Lui Y.-g., and Schmitt R.A. (1991) Paired lunar meteorites MAC88104 and MAC88105: a new “fan” of lunar petrology. Geochim Cosmochim Acta 55:3037–3049

[B66] Nishiizumi K., Arnold J.R., Kohl C.P., Caffee M.W., Masarik J., and Reedy R.C. (2009) Solar cosmic ray records in lunar rock 64455. Geochim Cosmochim Acta 73:2163–2176

[B67] Ogilvie K.W. and Coplan M.A. (1995) Solar wind composition. Rev Geophys 33:615–622

[B68] Ong L., Asphaug E.I., Korycansky D., and Coker R.F. (2010) Volatile retention from cometary impacts on the Moon. Icarus 207:578–589

[B69] Owen M.R. (1988) Radiation-damage halos in quartz. Geology 16:529–532

[B70] Parnell J., Bowden S., Lindgren P., Burchell M., Milner D., Price M., Baldwin E.C., and Crawford I.A. (2010) The preservation of fossil biomarkers during meteorite impact events: experimental evidence from biomarker-rich projectiles and target rocks. Meteorit Planet Sci 45:1340–1358

[B71] Rao M.N., Garrison D.H., Bogard D.D., and Reedy R.C. (1994) Determination of the flux and energy distribution of energetic solar protons in the past 2 Myr using lunar rock 68815. Geochim Cosmochim Acta 58:4231–4245

[B72] Reedy R.C., Arnold J.R., and Lal D. (1983) Cosmic-ray record in Solar System matter. Science 219:127–135 17841664 10.1126/science.219.4581.127

[B73] Rumpf M.E., Fagents S.A., Crawford I.A., and Joy K.H. (2013) Numerical modeling of lava-regolith heat transfer on the Moon and implications for the preservation of implanted volatiles. J Geophys Res: Planets 118:382–397

[B74] Sagan C. (1972) The search for indigenous lunar organic matter. Space Life Sci 3:484–489 4650303 10.1007/BF00926778

[B75] Schmitz B., Tassinari M., and Peucker-Ehrenbrink B. (2001) A rain of ordinary chondritic meteorites in the early Ordovician. Earth Planet Sci Lett 194:1–15

[B76] Schultz P.H. and Gault D.E. (1975) Seismically induced modification of lunar surface features. In Proceedings of the 6^th^ Lunar Science Conference, Vol. 3, Pergamon Press, New York, pp 2845–2862

[B77] Schwadron N.A., Baker T., Blake B., Case A.W., Cooper J.F., Golightly M., Jordan A., Joyce C., Kasper J., Kozarev K., Mislinski J., Mazur J., Posner A., Rother O., Smith S., Spence H.E., Townsend L.W., Wilson J., and Zeitlin C. (2012) Lunar radiation environment and space weathering from the Cosmic Ray Telescope for the Effects of Radiation (CRaTER). J Geophys Res Planets 117, doi:10.1029/2011JE003978

[B78] Sephton M.A. (2002) Organic compounds in carbonaceous meteorites. Nat Prod Rep 19:292–311 12137279 10.1039/b103775g

[B79] Spence H.E., Case A.W., Golightly M.J., Heine T., Larsen B.A., Blake J.B., Caranza P., Crain W.R., George J., Lalic M., Lin A., Looper M.D., Mazur J.E., Salvaggio D., Kasper J.C., Stubbs T.J., Doucette M., Ford P., Foster R., Goeke R., Gordon D., Klatt B., O'Connor J., Smith M., Onsager T., Zeitlin C., Townsend L.W., and Charara Y. (2010) CRaTER: The Cosmic Ray Telescope for the Effects of Radiation experiment on the Lunar Reconnaissance Orbiter mission. Space Sci Rev 150:243–284

[B80] Swallow A.J. (1960) Radiation Chemistry of Organic Compounds, Pergamon Press, New York

[B81] Taylor G.J., Warren P., Ryder G., Delano J., Pieters C.M., and Lofgren G. (1991) Lunar rocks. In The Lunar Sourcebook: A User's Guide to the Moon, edited by Heiken G.H., Vaniman D.T., and French B.M., Cambridge University Press, Cambridge, UK, pp 183–284

[B82] Thomas-Keprta K.L., Clemett S.J., Messenger S., Ross D.K., Le L., Rahman Z., McKay D.S., Gibson E.K. Jr., Gonzalez C., and Peabody W. (2014) Organic matter on the Earth's Moon. Geochim Cosmochim Acta 134:1–15

[B83] Tripathi R.K., Wilson J.W., Badavi F.F., and De Angelis G. (2006) A characterization of the Moon radiation environment for radiation analysis. Adv Space Res 37:1749–1758

[B84] Vaniman D., Reedy R., Heiken G., Olhoeft G., and Mendell W. (1991) The lunar environment. In The Lunar Sourcebook: A User's Guide to the Moon, edited by Heiken G.H., Vaniman D.T., and French B.M., Cambridge University Press, Cambridge, UK, pp27–60

[B85] Willman B.M., Boles W.W., McKay D.S., and Allen C.C. (1995) Properties of lunar soil simulant JSC-1. J Aerosp Eng 8:77–87

[B86] Wood B.E., Müller H.R., Zank G.P., Linsky J.L., and Redfield S. (2005) New mass-loss measurements from astrospheric Lyα absorption. Astrophys J 628, doi:10.1086/432716

[B87] Xiao Z., Zeng Z., Ding N., and Molaro J. (2013) Mass wasting features on the Moon—how active is the lunar surface? Earth Planet Sci Lett 376:1–11

[B88] Yamashita N., Hasebe N., Reedy R.C., Kobayashi S., Karouji Y., Hareyama M., Shibamura E., Kobayashi M.N., Okudaira O., d'Uston C., Gasnault O., Forni O., and Kim K.J. (2010) Uranium on the Moon: global distribution and U/Th ratio. Geophys Res Lett 37, doi:10.1029/2010GL043061

[B89] Zhang J.A. and Paige D.A. (2009) Cold-trapped organic compounds at the poles of the Moon and Mercury: implications for origins. Geophys Res Lett 36, doi:10.1029/2009GL038614

[B90] Zimmerman M.I., Farrell W.M., Stubbs T.J., Halekas J.S., and Jackson T.L. (2011) Solar wind access to lunar polar craters: feedback between surface charging and plasma expansion. Geophys Res Lett 38, doi:10.1029/2011GL048880

[B91] Zolensky M.E. (1997) Structural water in the Bench Crater chondrite returned from the Moon. Meteorit Planet Sci 32:15–18

